# Melatonin Modulates Cell Cycle Dynamics and Promotes Hippocampal Cell Proliferation After Ischemic Injury in Neonatal Rats

**DOI:** 10.1007/s12035-024-04013-x

**Published:** 2024-02-15

**Authors:** Barbara Canonico, Silvia Carloni, Mariele Montanari, Patrizia Ambrogini, Stefano Papa, Daniel Alonso-Alconada, Walter Balduini

**Affiliations:** 1 Department of Biomolecular Sciences, University of Urbino Carlo Bo, Via S. Chiara 27, 61029 Urbino, PU Italy; 2 Department of Cell Biology and Histology, School of Medicine and Nursing, University of the Basque Country (UPV/EHU), Leioa, Spain

**Keywords:** Neonatal ischemia, Melatonin, Flow cytometry, Cell cycle, Proliferation

## Abstract

Promoting neural cell proliferation may represent an important strategy for enhancing brain repair after developmental brain injury. The present study aimed to assess the effects of melatonin on cell proliferation after an ischemic injury in the developing hippocampus, focusing on cell cycle dynamics. After in vivo neonatal hypoxia–ischemia (HI), hippocampal cell cycle dynamics were assessed by flow cytometry, together with histological evaluation of dentate gyrus cellularity and proliferation. Melatonin significantly increased the number of proliferating cells in the G2/M phase as well as the proliferating cell nuclear antigen (PCNA) and doublecortin (DCX) labeling reduced by HI. In vivo BrdU labeling revealed a higher BrdU-positivity in the dentate gyrus of ischemic rats treated with melatonin, an effect followed by increased cellularity and preserved hippocampal tissue integrity. These results indicate that the protective effect of melatonin after ischemic injury in neonatal rats may rely on the modulation of cell cycle dynamics of newborn hippocampal cells and increased cell proliferation.

**Supplementary Information:**

The online version contains supplementary material available at 10.1007/s12035-024-04013-x.

## Introduction

Neurogenesis is the process by which new neurons are formed in the brain. Neurogenesis is crucial when an embryo is developing; it continues after birth and, in specific brain regions, it goes on throughout lifespan [1]. The timing of the cell proliferative processes in the immature brain varies between species, the different areas of the brain, and the cell phenotype. In rats, neurogenesis in cortical and subcortical regions begins during gestation and is completed at about postnatal day 15 [2, 3], with the majority of neuronal cells already present at birth. The dentate gyrus of the hippocampus, which forms prenatally, however, shows continued postnatal proliferation of granule cells [3]. Astrocytes, for their part, undergo proliferation and differentiation largely after neuronal cell differentiation and migration are completed; in rats, this occurs mostly postnatally up to postnatal day 14 [1]. Injurious events during the neonatal period, like hypoxia–ischemia (HI), could hinder these processes causing long-lasting deleterious effects in the neonate. In adult rats, ischemia modulates endogenous neurogenesis in two different ways. The first one is by the stimulation of migration of neural precursors to the lesioned area from the existing neurogenic niches, i.e., the subventricular zone (SVZ) bordering the lateral ventricles and the hippocampal subgranular zone (SGZ); these newborn cells are thought to participate in brain repair and functional recovery [4]. The second one concerns the local generation of new cells from areas close to the lesioned site, like the striatum, cortex, and hippocampus [5, 6]. However, despite the newly generated cells targeted to the injured site, their number can be insufficient to replace the injured ones or cannot be fully integrated in the new cytoarchitecture [7]. Differently from adults, developmental compensatory mechanisms in neonates may promote neural plasticity after brain injury allowing a better replacement and integration of the new cells [8]. Thus, promoting neural cell proliferation may represent an important strategy for enhancing brain repair after developmental brain injury.

There is plenty of literature showing that melatonin, a hormone synthesized in the pineal gland and other cells of the body that regulates a variety of physiological functions including circadian rhythms and neuroimmunity [9, 10], is neuroprotective after neonatal brain injury [11–13]. Besides reducing cell death, melatonin may also enhance brain repair by favoring cell proliferation and differentiation. In adult mice, for example, SVZ precursor cells express melatonin receptors and exhibit increased proliferative activity after melatonin treatment [14, 15], an effect also observed in the SGZ [16].

This study aimed to assess the early effects of melatonin on hippocampal cell cycle dynamics and cellularity after an ischemic injury during brain development in neonatal rats. After in vivo neonatal HI, cell cycle and proliferation were assessed by flow cytometry in the whole hippocampal tissue 1 h after the ischemic insult, followed by immunohistochemical studies in the medium term.

## Material and Methods

All surgical and experimental procedures were carried out in accordance with the Italian regulation for the care and use of laboratory animals (EU Directive 63/2010; Italian D.L. 26/14; research protocol authorization 582/2020-PR) and were approved by the Animal Care Committee of the University of Urbino Carlo Bo.

### In vivo* Cerebral Hypoxia–Ischemia (HI)*

Pregnant Sprague–Dawley rats were housed in individual cages, and the day of delivery was considered day 0. Neonate rats from different litters were randomized, normalized to ten pups per litter, and kept in a regular light/dark cycle (lights on 6 am–6 pm). On postnatal day (PN) 7, after anesthesia with 5% isoflurane in O_2_, rat pups underwent unilateral ligation of the right common carotid artery via a midline neck incision. After artery ligation, the wound was sutured, and the animals were allowed to recover for 3 h with their dams. Pups were then placed in an airtight jar and exposed for 2.5 h to a humidified nitrogen–oxygen mixture (92% and 8%, respectively) delivered at 5–6 L/min to induce hypoxia. The jar was partially submerged in a 37 °C water bath to maintain a constant thermal environment. Once the HI procedure was finished (and melatonin treatment or vehicle received), pups were returned to their dams until the experimental procedures were performed at 1 h, 72 h (PN10), or 7 days (PN14) after HI.

### Drug Treatment

Melatonin (Sigma-Aldrich, Milan, Italy, M525), dissolved in dimethyl sulfoxide (DMSO) and diluted in normal saline solution to a final concentration of 5% DMSO (vehicle), was intraperitoneally injected to pup rats 5 min after HI at a dose of 15 mg/kg and repeated after 24 h and 48 h (HI + MEL, *N* = 15). Control sham-operated (Sham, *N* = 15) and hypoxic-ischemic (HI, *N* = 15) animals received the same volume of vehicle. The dose of melatonin was chosen on the basis of previous experiments showing the protective effects of melatonin in ischemic neonatal pup rats [13, 17].

### Brain tissues disaggregation

We used a mechanical disaggregation method to obtain functional cells from neonatal hippocampal tissue [18, 19]. Briefly, pup rats were anesthetized and euthanized by decapitation 1 h after HI (Sham, HI, HI + MEL, *N* = 5 per group). Brains were rapidly removed, dissected on ice, and the hippocampus isolated. Homogenates from each experimental sample were prepared using the Medimachine System (CTSV s.r.l.; Brescia, Italy) at a constant speed of 100 rpm for a time ranging from 15 to 25 s. The cell suspension was then aspirated with a syringe from the bottom part of the Medicon capsule. After the first run, 1 mL of PBS was again added to the processed remaining tissue for 10 s, and the cell fractions were subsequently pooled. Finally, the cell suspension was filtered using a 70 µm Filcon (first passage) and a 50 µm Filcon (second passage) (CTSV s.r.l.; Brescia, Italy).

### DNA content and cell cycle evaluation

Cellular suspension, obtained after disaggregation with Medimachine II as described above, was fixed with cold ethanol (70%, − 20 °C) and stored at + 4 °C. At the time of analysis, samples were washed twice with PBS, and the cell pellet was resuspended in PBS containing propidium iodide (PI, 1 mg/mL; P4170, Sigma-Aldrich) and RNAse (1 mg/mL; 10,109,142,001, Sigma-Aldrich). Proliferating cell nuclear antigen (PCNA) was studied using an anti-PCNA FITC-conjugated antibody (1:200; CBL407, clone PC10, Sigma-Aldrich). After incubation in thermostatic conditions (37 °C) for at least 30 min, samples were analyzed for cell cycle on the FACSCanto II cytometer (BD) equipped with an argon laser (Blue, Ex 488 nm), a helium–neon laser (Red, Ex 633 nm), and a solid-state diode laser (Violet, Ex 405 nm). The analyses were performed with the FACSDiva™ (BD) software. Approximately 10,000 cell events were acquired for each sample.

### Detection of doublecortin labeling

The fixed disaggregated cells were incubated with the anti-doublecortin antibody (anti-DCX; 1:200, rabbit, polyclonal; sc-8066, Santa Cruz Biotechnology) for 45 min at room temperature. After washing, the cells were incubated for 30 min at room temperature with a goat FITC-conjugated anti-rabbit secondary antibody (1:50 in PBS, D.B.A) and processed for flow cytometric analysis. All cytometric experiments were carried out with a FACSCanto II flow cytometer (BD, Franklin, Lakes, NJ, USA) equipped with an argon laser (Blue, Excitation 488 nm), a helium–neon laser (Red, Excitation 633 nm), and a solid-state diode laser (Violet, Ex 405 nm). Analyses were performed with the FACSDiva™ software (BD); approximately 10,000 cell events were acquired for each sample.

### Histology

#### Tissue Processing

On PN14 (7 days after HI), another group of animals was euthanized via lethal injection of sodium pentobarbital and perfusion-fixed with 4% paraformaldehyde in 0.1 mol/L PBS. Brains were rapidly removed and post-fixed at 4 °C overnight. Tissue blocks were then prepared for paraffin embedding.

#### Hippocampal Ratios

At the level of mid-dorsal hippocampus and thalamus (Bregma -1.80 mm) [20], 5-µm paraffin-embedded brain slices were obtained and processed for Harris’ hematoxylin and eosin (H&E) staining. Using an Olympus BX40 light microscope, H&E-stained slices were analyzed by two researchers blinded to the treatment group. From × 4 magnification microphotographs, both ipsilateral (damaged) and contralateral (non-damaged) hippocampal areas were outlined, and the ratio of ipsi- to contralateral areas was calculated using Fiji/ImageJ image software.

#### Dentate Gyrus Cell Counts

Using the same H&E-stained samples as for hippocampal ratios, the number of cells was counted in the dentate gyrus of the hippocampus. In each sample, 8 non-overlapping microphotographs (4 in each hippocampus) were taken at × 40 magnification (high-power-field or HPF), and the total number of morphologically well-preserved cells was counted using Fiji/ImageJ image software.

### BrdU Uptake and Labeling

To determine the proliferation rate of newborn cells, we applied the standard methodology of cell incorporation of 5-Bromo-2′-deoxyuridine (BrdU), which is incorporated into the DNA of dividing cells instead of thymidine and labels proliferating cells. In vivo BrdU labeling was performed in additional groups of Sham, HI, and HI + MEL animals (*N* = 5 per group). BrdU (100 mg/Kg, B5002, Sigma-Aldrich) was administered through intraperitoneal injection to PN9 neonatal rats (i.e., 48 h after HI), and 24 h later (PN10), animals were euthanized and perfusion-fixed with 4% paraformaldehyde in 0.1 mol/L PBS [21].

Immunohistochemical BrdU staining was performed as previously reported [22]. Briefly, in brain slices (thickness 20 µm), DNA was denatured with 2 M HCl for 30 min at 37 °C and after neutralizing for 10 min in 0.1 M sodium borate buffer (pH = 8.5), slices were permeabilized with TBS-plus (TBS containing 1% triton-X 100 and 1.5% goat serum) for 1 h at room temperature, and then overnight at 4 °C with the anti-mouse BrdU antibody (1:200; monoclonal; #5292, Cell Signaling Technology). The secondary antibody biotinylated goat anti-mouse (1:200; B7264, Sigma-Aldrich) was visualized using the Elite ABC kit (VECTASTAIN® ABC-HRP Kit, Peroxidase; PK-4000). Peroxidase activity was revealed by 0.05% DAB and 0.03% H_2_O_2_ at the appropriate stage. The reaction specificity was evaluated in some slices by omitting the primary antibody from the incubation medium.

Cell counting of BrdU-positive cells was conducted in the dentate gyrus of the hippocampus on × 20 microscopic images using a BX-51 Olympus microscope (Olympus Italia S.r.l., Milan, Italy). Positive cells were counted using the NIH-ImageJ 1.45 software (https://imagej.nih.gov/ij/, National Institutes of Health, Bethesda, MD, USA) in four separate fields of the dentate gyrus in slices cut at the level A 3750 of the Koning and Klippel stereotaxic atlas [23]. Five animals for each group were analyzed.

### Statistical Analysis

Analysis of variance (ANOVA) approaches were used to compare values among more than two different experimental groups for data that met the normality assumption. One-way ANOVA or two-way ANOVA were followed by a Bonferroni posthoc test or Dunnett’s multiple comparison test. A *p*-value < 0.05 was considered significant. All statistical analyses were performed using GraphPad Prism 9.0 (GraphPad Software, San Diego, CA, USA).

## Results

### Melatonin Rapidly Primed Cell Proliferation in the Ischemic Hippocampus of Neonatal Rats

**Fig. 1:**
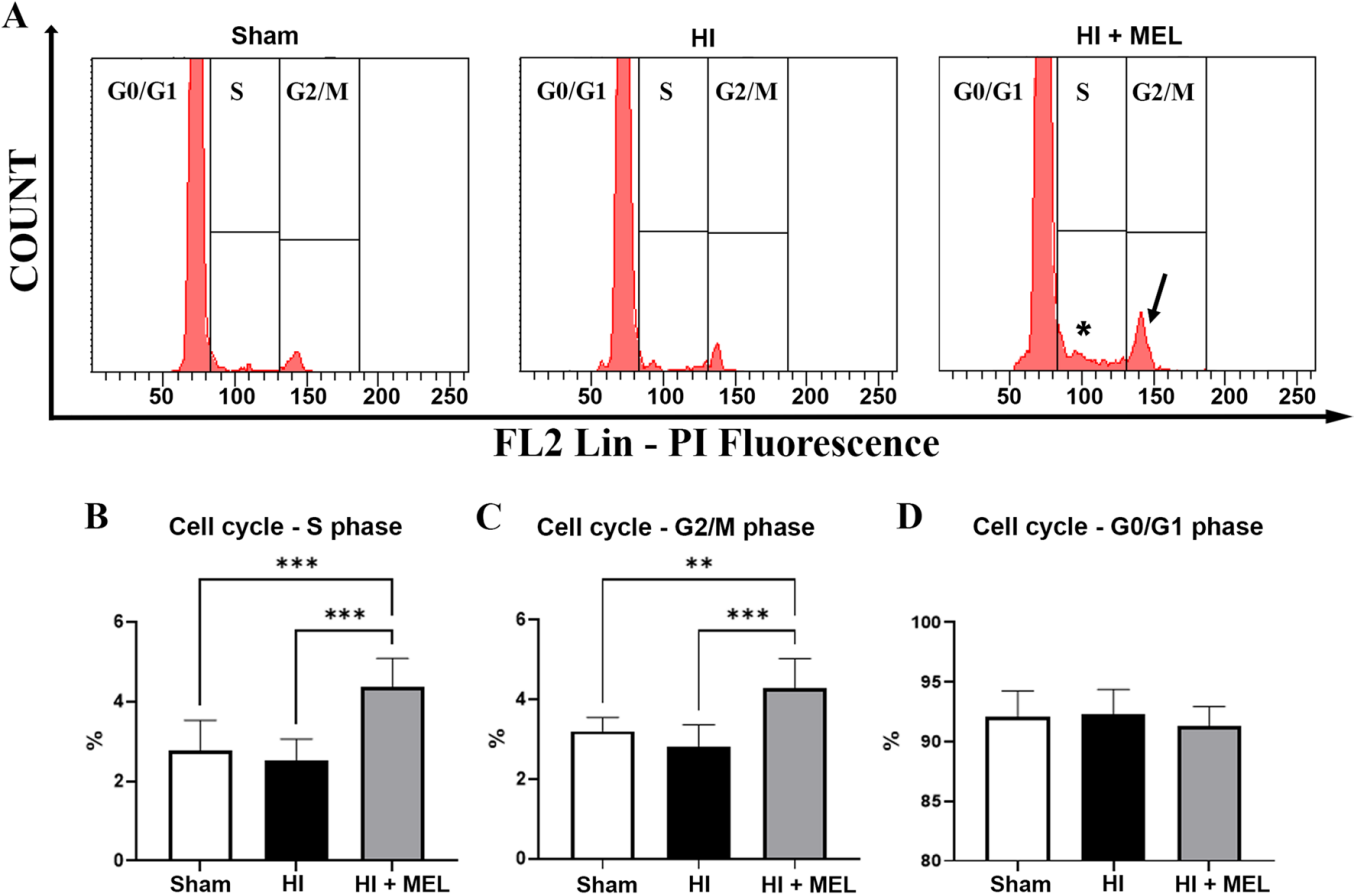
**A** Flow cytometric profiles for DNA content evaluation in cells from the hippocampus of control sham-operated animals (Sham) and of vehicle-treated (HI) or melatonin-treated ischemic animals (HI + MEL). The asterisk and arrow highlight the area of DNA synthesis increasing and G2/M peak increase in HI + MEL animals, respectively. **B** Flow cytometric analysis of cell cycle S phase, G2/M phase (**C**), and G0/G1 phase (**D**) in cells from the hippocampus of Sham, HI, and HI + MEL animals. Results are expressed as a percentage of all acquired events. Values represent the mean ± SD of cell events acquired in 3 hippocampi for each experimental condition. Each measurement was performed in triplicates. **p < 0.01, ***p < 0.001, two-way ANOVA with a Bonferroni’s multiple comparison test

The effect of HI and melatonin administration on the cell cycle was assessed after isolation of the hippocampus in vivo and its mechanical disaggregation. Exclusion of doublets was performed for an accurate evaluation of G2/M events (Fig. S1 A). HI did not significantly affect the percentage of cells in the S phase and those in the G2/M phase (Fig. 1B and C), although a trend toward reduction was observed in HI animals compared to Sham (Fig. 1A and B). Melatonin increased the percentage of cells in the S phase (Fig. 1A, asterisk; Fig. 1B) and in the G2/M phase (Fig. 1A, arrow; Fig. 1C). Neither HI nor melatonin significantly affected the percentage of cells in the G0/G1 phase (Fig. 1D). Although the S and G2/M phases, separately reported, better described the priming of proliferation [24], the percentages of overall proliferating cells are reported in Figure S1, panel B.

**Fig. 2:**
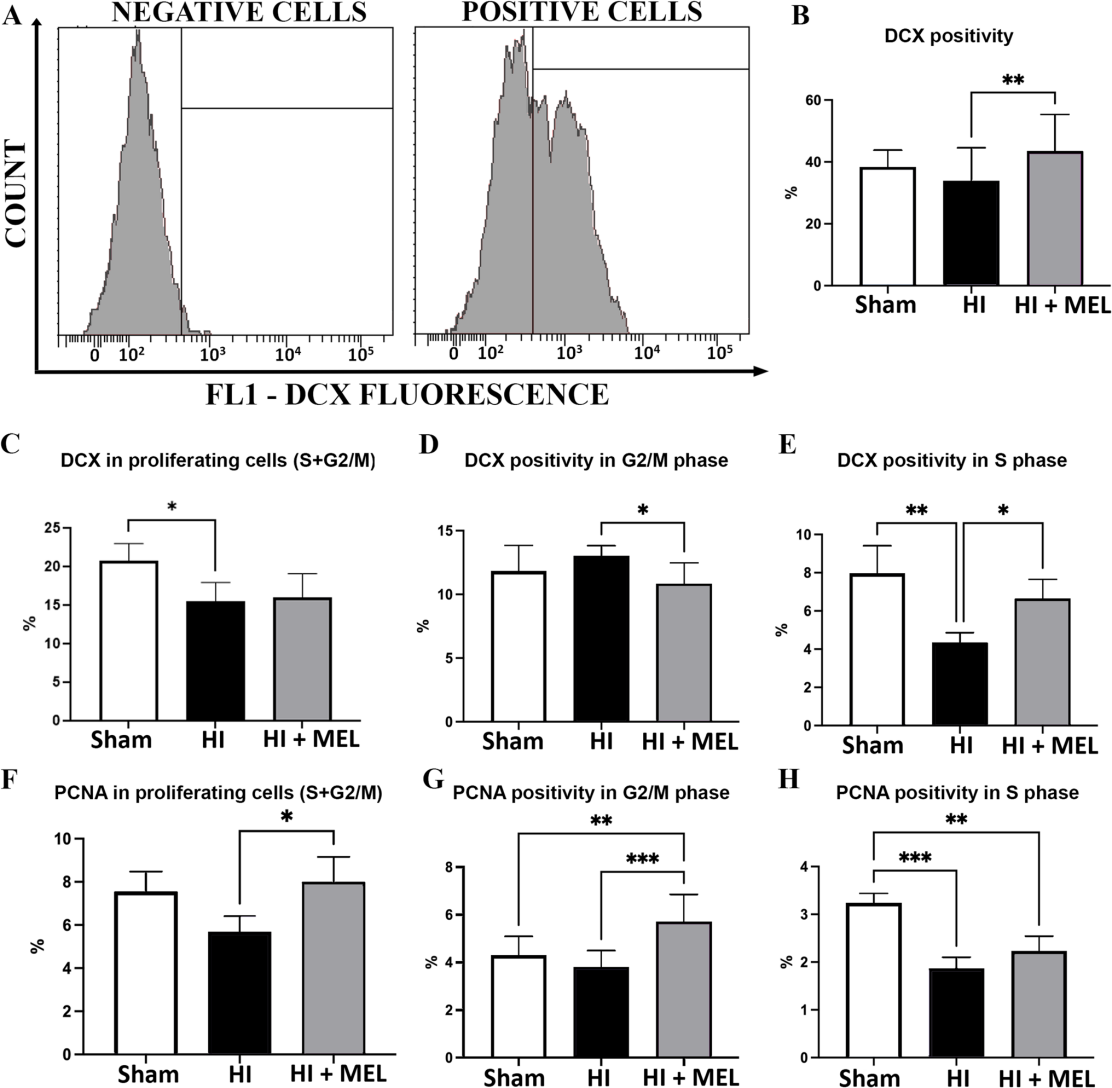
**A** Representative cytometric histogram of doublecortin (DCX) positive:negative control cells (to establish the threshold for fluorescence positivity) and DCX-labeled cells. **B** Quantitative evaluation of DCX labeling in total cells (**B**), S/G2M phase cells (**C**), G2/M phase cells (**D**), and S phase cells (**E**) of the hippocampus of control sham-operated animals (Sham), vehicle-treated (HI), or melatonin-treated ischemic animals (HI + MEL). **F** Quantitative evaluation of proliferating cell nuclear antigen (PCNA) labeling in S/G2M phase cells, G2/M phase cells (**G**), and S phase cells (**H**) of the hippocampus of Sham, HI, and HI + MEL animals. Results are expressed as a percentage of all acquired events. Values represent the mean ± SD of cell events acquired in 3 hippocampi for each experimental condition. Each measurement was performed in triplicates. *p < 0.05, **p < 0.01, ***p < 0.001, Two-way ANOVA with a Bonferroni’s multiple comparison test

We then evaluated the expression of DCX, an essential factor in neurogenesis [25], and PCNA, which is a well-known molecular marker for cell proliferation directly involved in DNA repair [26], in the whole hippocampus after mechanical disaggregation. Flow cytometric analysis was performed on ethanol-fixed samples, and the specificity of DCX labeling was verified (Fig. 2A). No significant effect of HI was observed 1 h after the insult on DCX labeling (Fig. 2B), whereas melatonin significantly increased the percentages of DCX events compared to HI (Fig. 2B). Interestingly, the flow cytometric analysis of DCX expression performed during the cell cycle progression revealed a mild but significant reduction in proliferating (S + G2/M) cells in the ischemic hippocampus 1 h after injury (Fig. 2C), with a peculiar distribution in G2/M (Fig. 2D) and S phase cells (Fig. 2E). These effects induced by HI were hampered in the hippocampus of melatonin-treated ischemic animals (Fig. 2C–E). HI also affected PCNA expression assessed during the cell cycle progression (Fig. 2F–H). One hour after HI, PCNA expression was significantly reduced in the proliferating (S + G2/M) hippocampal cells (Fig. 2F) as well as in cells in the S phase (Fig. 2H). Melatonin hampered the HI-induced reduction of PCNA + cells in total proliferating hippocampal cells (S + G2/M, Fig. 2F), highlighting intriguing differences in S and G2/M phases (Fig. 2H). Interestingly, melatonin increased the PCNA positivity in G2/M cells over control levels (Fig. 2G).

### Melatonin Preserved Hippocampal Integrity and Cellularity and Promoted Cell Proliferation in the Ischemic Dentate Gyrus of Neonatal Rats

**Fig. 3:**
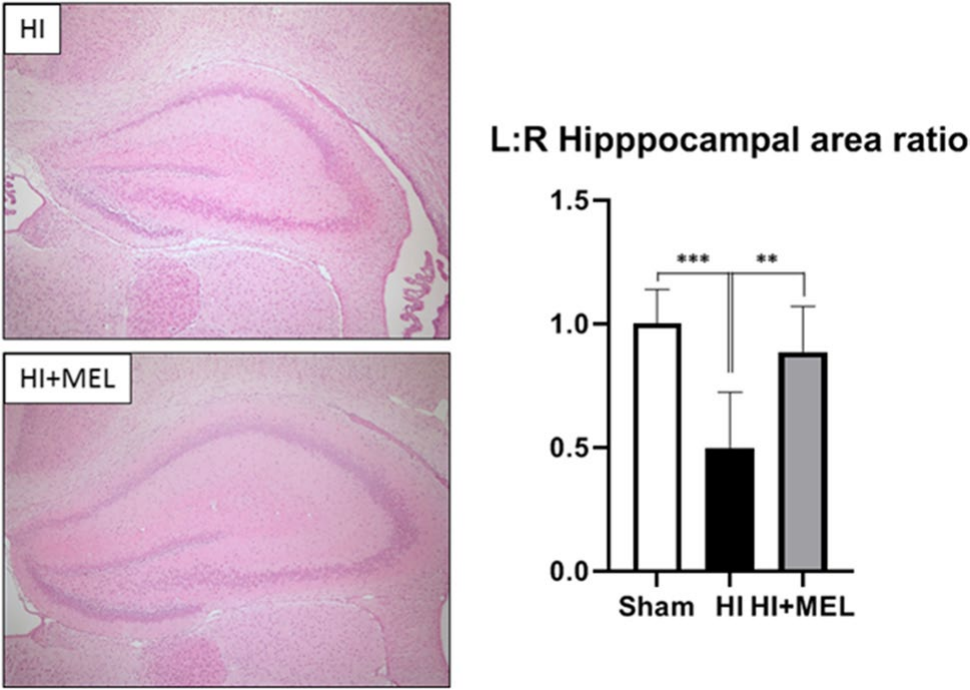
Area of the hippocampus. Representative microphotographs of the ipsilateral (damaged/ischemic) hippocampus from vehicle-treated (HI) and melatonin-treated (HI + MEL) animals 7 days after HI injury (PN14). H&E staining. Magnification, × 4. Graph: hippocampal area data was expressed as right-to-left (ipsilateral-to-contralateral) ratio from Sham, HI, and HI + MEL treated animals. HI induced a reduction in the area of the right (ipsilateral) hippocampus, thus significantly (***p < 0.001 vs Sham) decreasing the hippocampal area ratio. Melatonin administration (HI + MEL) showed a higher hippocampal area ratio when compared to HI (**p < 0.01), obtaining similar values to those observed for Sham animals. One-way ANOVA followed by the Dunnett’s multiple comparison test (*N* = 5 animals/group)

**Fig. 4:**
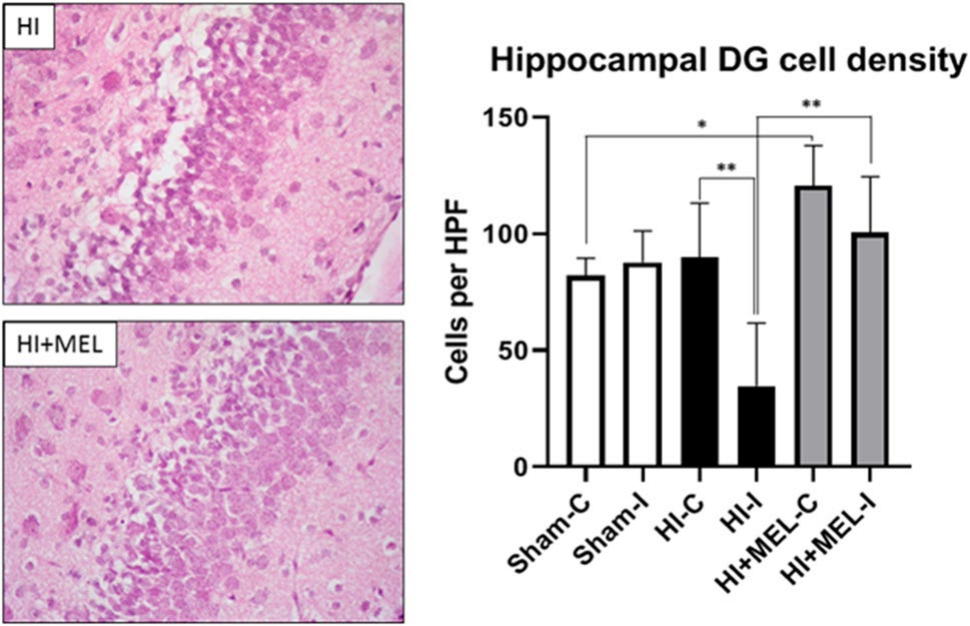
Cellularity of the hippocampal dentate gyrus. Representative microphotographs of the ipsilateral (damaged/ischemic) dentate gyrus of the hippocampus from vehicle (HI) and melatonin-treated (HI + MEL) animals 7 days after HI injury (PN14). H&E staining. Magnification, × 40. Graph: Hippocampal dentate gyrus cellularity was expressed as the number of cells per high-power field (40 × microphotograph) from Sham, HI, and HI + MEL animals. HI induced a significant decrease in the cellularity of the ipsilateral (I, ipsilateral side to the occluded carotid artery) dentate gyrus compared to the contralateral one (C, contralateral side to the occluded carotid artery) (**p < 0.01, HI-I vs HI-C). Melatonin administration after HI showed higher cell counts in the ipsilateral (damaged) dentate gyrus (**p < 0.01, HI-I vs HI + MEL-I). This effect was also extended to the contralateral (non-ischemic) dentate gyrus (**p < 0.01, HI + MEL-C vs Sham-C). One-way ANOVA followed by the Dunnett’s multiple comparison test (*N* = 5 animals/group)

The experiments reported above were performed 1 h after in vivo HI-induced injury and melatonin administration. To assess if the priming effect of melatonin resulted in increased cellularity, we evaluated the effect of in vivo HI and melatonin treatment on hippocampal tissue integrity and dentate gyrus cellularity 7 days after the injury (PN14). Quantitative evaluation of the hippocampal tissue area revealed a significant reduction of the ipsilateral (damaged/ischemic) hippocampal area after HI, an effect that was preserved in melatonin-treated animals (Fig. 3), in agreement with previous data [12, 13]. We then analyzed the cellularity of the dentate gyrus (the neurogenic niche of the hippocampus) from both contralateral (non-damaged) and ipsilateral (damaged) hippocampi (Fig. 4). In the contralateral dentate gyrus, we found no differences in cell counts between Sham and HI animals, whereas in melatonin-treated animals, the number of cells was significantly higher compared to sham animals. In the ipsilateral (damaged) hippocampus, HI-induced reduction in the cellularity of the dentate gyrus was completely reverted by melatonin administration (Fig. 4; **p < 0.01, HI-I vs HI + MEL-I), thus obtaining values similar to Sham animals.

**Fig. 5:**
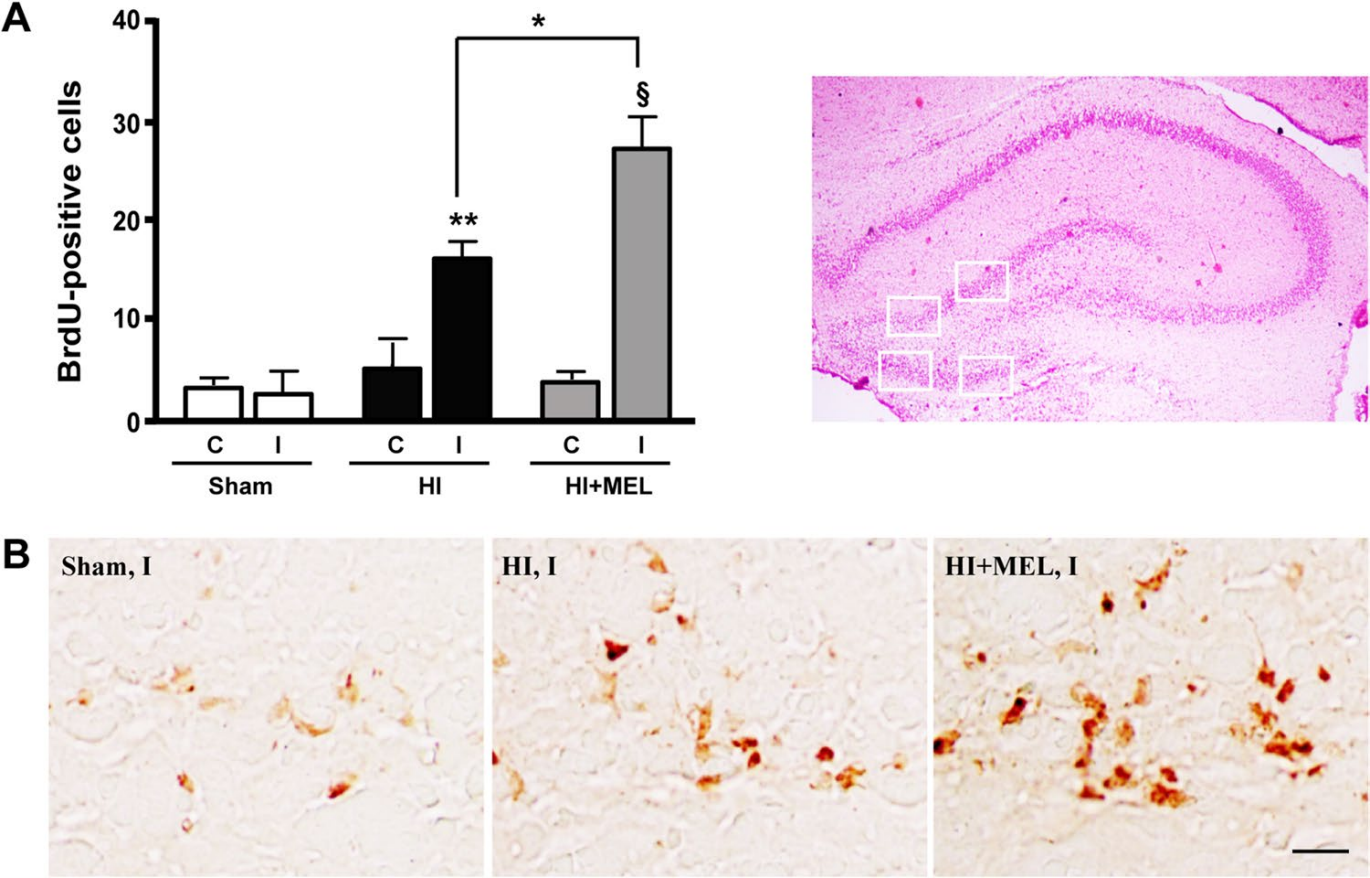
**A** Counts of BrdU-positive cells in the hippocampus of control sham-operated animals (Sham), vehicle-treated (HI), and melatonin-treated (HI + MEL) ischemic animals. BrdU was administered to postnatal 9 (PN9) rats, i.e., 48 h after HI, and sacrificed at PN10. Counts of BrdU-positive cells were performed as described in the “Materials and Methods” section, and data were reported as mean ± SE. The upper right part of the figure shows the selected areas of the dentate gyrus of the hippocampus analyzed for BrdU-positive cell counting. C and I, contralateral and ipsilateral side to the occluded carotid artery, respectively. *p < 0.05, **p < 0.01, §*p* < 0.001, One-way ANOVA followed by Dunnett’s multiple comparison test (*N* = 5 animals/group). **B** Representative microphotograph showing BrdU-positive cells in the ipsilateral hippocampus of Sham, HI, and HI + MEL animals. Scale-bar, 50 µm

To confirm that the effect of melatonin on dentate gyrus’ cellularity could also be ascribed to its ability to stimulate cell proliferation, we performed in vivo BrdU labeling. Few BrdU-positive cells were observed in the dentate gyrus of Sham animals as well as in the contralateral (non-damaged) hippocampi of HI and HI + MEL animals (Fig. 5A). BrdU labeling significantly increased in the ischemic (ipsilateral) hippocampus 3 days after HI injury, i.e., PN10 (Fig. 5A and B). Melatonin further increased the number of BrdU-positive cells in the ipsilateral dentate gyrus compared to those found in the HI group (Fig. 5A and B).

## Discussion

Neuroplasticity represents an essential feature of the developing central nervous system, which changes its structure, functions, connections, or activity in response to intrinsic or extrinsic stimuli or damaging events. After an ischemic event leading to cell death, the brain may favor the formation of new cells and connections for neuroregeneration [27]. These new cells can provide new resources in the long term to replace damaged cells and achieve a more-or-less complete functional recovery. This may depend on the severity of the injury and the developmental stage of Melatonin significantly increased DCX positivity, peculiarly affecting cells in the S phase. To our knowledge, this is the first study assessing DCX expression during the different phases of the cell cycle in immature animals at an early time after the ischemic lesion. Our data are in line with the modulation by melatonin of neural stem cells [38] and precursor forebrain maturation at which the injury occurs. Neuroprotective drugs mainly target the neurodegenerative process but may also influence regeneration and plasticity; these combined effects may favor brain recovery. Here, we used hippocampal tissue obtained from an in vivo model of neonatal HI to assess the potential ability of melatonin to stimulate cell proliferation and influence the regenerative process. To this end, we used a mechanical method of brain tissue disaggregation [18] to analyze hippocampal cells by flow cytometry. The mechanical disaggregation method allows the analysis of cellular proliferation, maintaining the transcriptome and proteotype of both neuronal and glial cells that, instead, could be altered by standard enzymatic digestion [19]. Results showed that HI did not significantly affect the percentage of cells in the S phase and G2/M phase. Melatonin significantly increased the number of proliferating cells in both the S and G2/M phases, suggesting that it can prime cells toward proliferation. Immunohistochemical experiments showed increased BrdU positivity in the ischemic dentate gyrus of the hippocampus of HI animals. These data are in keeping with previous results on the effects of ischemia on cell proliferation and differentiation. Indeed, it has been shown that neonatal ischemia-induced brain injury activates endogenous neurogenesis by itself, resulting in partial injury recovery [21, 28]. Our data showing a priming effect of melatonin supports the idea that neuroprotective agents may also stimulate neuroregeneration besides reducing cell death. In line with this hypothesis, BrdU-uptake experiments showed that melatonin improved the proliferative potential of the surviving cells in the injured area. Indeed, melatonin further increased BrdU positivity in the ipsilateral dentate gyrus of the hippocampus at PN10 and increased cellularity at PN14. The latter findings are in accordance with previous studies [29, 30]. A modulating effect of melatonin on cell cycle regulatory proteins has been recently reported on the striatum in adult mice who underwent focal cerebral ischemia induced by middle cerebral artery occlusion (MCAO) [31]. We speculate that the increased cellularity observed in the hippocampus after melatonin treatment could be due, at least in part, to its priming effect on cell proliferation in the dentate gyrus [32].

The effects of melatonin paralleled an increased DCX and PCNA labeling. DCX is a microtubule-associated protein expressed in the neuronal progenitors and early immature neurons [33, 34]. DCX is expressed in a precise temporal manner in migrating neuroblasts during early embryonic development and in neurogenic niches in adults, i.e., the SVZ and SGZ of the dentate gyrus of the hippocampus [35, 36]. Our flow cytometry analysis showed DCX expression in proliferating cells (S + /G2M) in the hippocampus, indicating that assessment of DCX by flow cytometric analysis in ethanol-fixed cell samples may represent a sensible method for monitoring the early expression of the protein during the cell cycle progression in vivo [37]. Our experiments also showed that HI significantly reduced DCX expression in the early phase of brain injury. Melatonin significantly increased DCX positivity, peculiarly affecting cells in the S phase. To our knowledge, this is the first study assessing DCX expression during the different phases of the cell cycle in immature animals at an early time after the ischemic lesion. These data are in line with the modulation by melatonin of neural stem cells [38] and precursors from the adult mouse SVZ, which represents the main neurogenic area of the adult brain [15]. The absence of increased DCX events in the G2/M selected cellular pool from melatonin-treated ischemic hippocampus is physiologic and expected in this early time point of investigation. Considering that the examined cells are not synchronized and that melatonin can infer on those DCX + cells already in the G2/M phase during its addition, we speculate that melatonin could prime their exit from the cell cycle, with the possible goal to re-enter and/or differentiate and then replace cell loss after ischemic injury, as revealed by histologic analyses.

DNA integrity is checked before mitosis, and if damage is detected, the cell cycle is halted, and apoptosis may occur [39]. PCNA expression, which is required for both DNA replication and DNA repair [26, 40], is normally up-regulated in proliferating cells during the S phase of the cell cycle [41] and may represent a potential marker of protection in ischemic conditions [42, 43]. Our results show increased PCNA positivity in S-phase cells, and its upregulation over control levels G2/M phase cells further supports the priming effect of melatonin on cell proliferation and neuroprotection. In keeping with our findings, it has been reported that PCNA was modulated by melatonin after transient middle cerebral artery occlusion in adult rats [42]. In addition, Li and coworkers showed that upregulation of the expression of PCNA via the p53 pathway reduced neuronal cell death [43].

In summary, our findings show that the protective effect of melatonin after ischemic hippocampal injury in neonatal rats may be related, at least in part, to the modulation of the cell cycle dynamics and increased cell proliferation of newborn hippocampal cells. Our results indicate that melatonin primes survival cells to proliferation, favoring an early replacement of damaged cells in the infarcted area. To our knowledge, this is the first study addressing in vivo cell cycle dynamics after a neuroprotective dose of melatonin in newborn rats. Nevertheless, this study presents some limitations since it did not identify the type of cells primed by melatonin, its evolution over time, and its long-lasting effect on neurogenesis. Therefore, further studies are needed to clarify these issues as well as to identify the mechanisms through which melatonin can support these effects.

### Supplementary Information

Below is the link to the electronic supplementary material.

Supplementary file1 (TIF 83 KB)

## Data Availability

The datasets generated during and/or analyzed during the current study are available from the corresponding author upon reasonable request.
