# Supplementary figures and images for: Melatonin Modulates Cell Cycle Dynamics and Promotes Hippocampal Cell Proliferation After Ischemic Injury in Neonatal Rats
Source: Mol Neurobiol. 2024 Feb 15;61(9):6910–9. doi: 10.1007/s12035-024-04013-x (PMC11339182; doi:10.1007/s12035-024-04013-x)

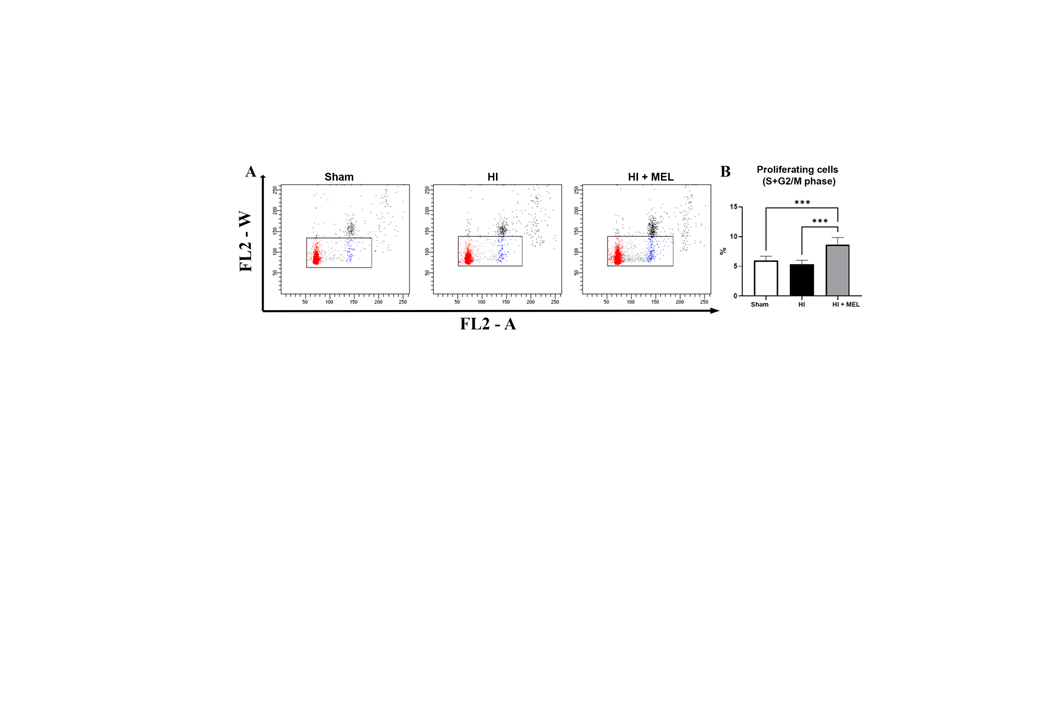

Supplement: Supplementary file 1 — Supplementary file1 (TIF 83 KB) [file 12035_2024_4013_MOESM1_ESM.tif]
